# Supplementary material for: Liver Transplantation for T2 Hepatocellular Carcinoma during the COVID-19 Pandemic: A Novel Model Balancing Individual Benefit against Healthcare Resources
Source: Cancers (Basel). 2021 Mar 19;13(6):1416. doi: 10.3390/cancers13061416 (PMC8003429; doi:10.3390/cancers13061416)
Supplement: Supplementary file 1 [file cancers-13-01416-s001.pdf]

Supplementary Table S1. Main characteristics of 8,567 Italian patients entering the waiting list for LT between January 2012 and December 2018. These variables were used to generate inverse probability of censoring weights to calculate median survival and median time to transplant of WL patients.

VARIABLES	N (%) or Median (IQR)
Age > 60 years old	2777 (32.4)
Male sex	6538 (76.3)
Body mass index > 25	4338 (52.2)
Viral aetiology of liver disease	4782 (55.9)
Blood group B-AB	1548 (18.1)
Stratification according to disease severity (%)	
MELD ≤ 20	3090 (36.7)
MELD 21-30	1120 (13.3)
MELD > 30	319 (3.8)
HCC	3897 (46.3)
MELD in non HCC patients	17 (14-22)
MELD in HCC patients	11 (8-14)
Donor age	63 (49-74)
Status at the end of follow-up	
Transplanted	6476 (75.6)
Dropped-out	1345 (15.7)
Still waiting	746 (8.7)

Abbreviations: MELD, model for end stage liver disease; HCC, hepatocellular carcinoma; LT, liver transplantation

Supplementary Table S2. Harm caused to individual patients on the waiting list when one organ is allocated. Usual and 50% decrease of organ arrival scenarios are represented.

Patient subgroup	Increase in mortality/dropout risk (per patient)	Days of life lost (per patient)
HCC		
- Usual organ arrival	0.4 %	5
- 50% acute decrease of organ availability	0.8 %	10
MELD ≤ 20		
- Usual organ arrival	0.3 %	4
50% acute decrease of organ availability	0.6 %	8
MELD 21-30		
- Usual organ arrival	2.3 %	34
- 50% acute decrease of organ availability	4.6 %	68
MELD > 30		
- Usual organ arrival	15.5 %	135
50% acute decrease of organ availability	31.0 %	270

Abbreviations: HCC, hepatocellular carcinoma; MELD, model for end stage liver disease

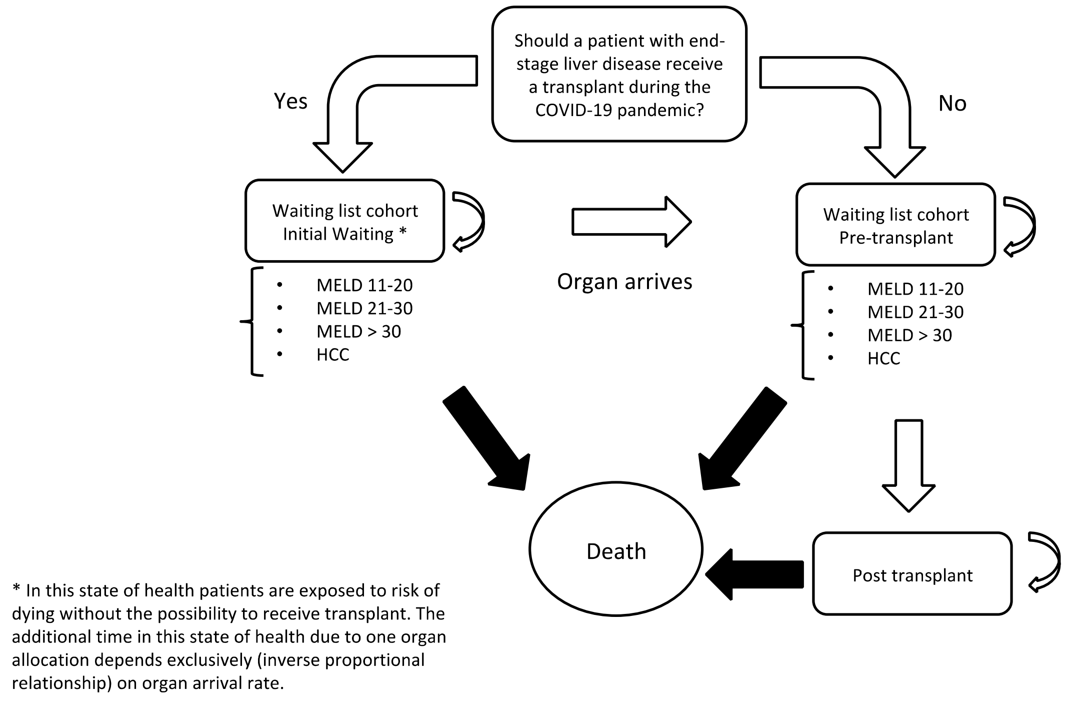

Supplementary Figure S1. **Influence diagram describing the structure of the model.**
If an organ is allocated, all the patients in the waiting list cohort start off in an initial waiting state and progress to pre-transplant state when the first organ arrives. If the organ is not allocated, then all the waiting list patients start off in the pre-transplant state. The solid black arrow indicates that death can occur from any state. The difference in intention to treat 5-year life expectancy between these two strategies (strategy No minus strategy Yes) was used to calculate the harm to others on the waiting list derived from individual allocation. In simple words, with strategy Yes WL patients will stay some additional days (depending on organ arrival rate) on the WL without the possibility to receive an organ; for this reason life expectancy of strategy Yes will be intrinsically lower than strategy No, and this loss in life expectancy represents the harm to others on the WL due to individual organ allocation.
